# Endothelium, Platelets, and Coagulation Factors as the Three Vital Components for Diagnosing Bleeding Disorders: A Simplified Perspective with Clinical Relevance

**DOI:** 10.1155/2022/5369001

**Published:** 2022-08-27

**Authors:** Abhinav Bhattarai, Sangam Shah, Sara Bagherieh, Omid Mirmosayyeb, Sangharsha Thapa, Sandip Paudel, Pawan Gyawali, Pitambar Khanal

**Affiliations:** ^1^Institute of Medicine, Tribhuvan University, Maharajgunj 44600, Nepal; ^2^Isfahan Neuroscience Research Center, Isfahan, Iran; ^3^Department of Neurology, Jacobs Comprehensive MS Treatment and Research Center, Jacobs School of Medicine and Biomedical Sciences, University, Buffalo, State University of NY, Buffalo, NY, USA

## Abstract

Bleeding disorders are a major group of hematological disorders, which are highly prevalent in the world. Excessive bleeding can result in serious consequences including hypoperfusion and cardiac arrest. The body has its selfmechanism to control excessive bleeding which is termed hemostasis. Hemostasis is achieved in two major steps, the formation of the primary and secondary hemostatic plugs. Endothelium, platelets, and coagulation factors are three components involved in hemostasis. Endothelium and platelets have a major role in forming the primary hemostatic plug. Consequently, the first step in investigating a bleeding disorder is platelet count. Despite normal platelet count, abnormality in the primary hemostatic plug may arise due to functional defects of the platelets including adhesion, activation, and aggregation. Von Willebrand disease (VWD) is an endothelial defect and the most prevalent inherited defect in coagulation. Abnormalities in the secondary hemostatic plug are largely due to coagulation factor deficiencies, and, to a lesser extent, the presence of inhibitors. Techniques involving viscoelastics have been aiding in rapid diagnosis and are useful in point-of-care testing. This article discusses the investigation of bleeding disorders from the perspective of the endothelium, platelet, and coagulation factor physiology. These three components should be properly investigated to achieve the definitive diagnosis of bleeding disorders.

## 1. Introduction

Bleeding is the spontaneous escape of blood from the blood vessels following trauma. Excessive loss of blood from the vessels may lead to serious consequences including reduced blood pressure, tissue hypoperfusion, and cardiac arrest. Owing to the body for its innate mechanism for controlling excess bleeding, such consequences seldom occur [1]. The mechanism by which the body renders the bleeding concluded is hemostasis. Hemostasis is accomplished in four distinct stages, starting from blood vessel constriction, formation of the platelet plug, formation of a stable insoluble fibrin clot, and finally removal of the fibrin clot [2]. Following bleeding, there is vasoconstriction, followed by adhesion, activation, and aggregation of platelets, occluding the site of bleeding. This process is referred to as the primary hemostatic plug formation. The coagulation factors are then activated, forming thrombin, which in turn forms insoluble fibrin from fibrinogen. Afterward, Factor XIII, which is activated by thrombin, strengthens the fibrin clot by cross-linkage, forming the secondary hemostatic plug [3].

**Figure 1:**
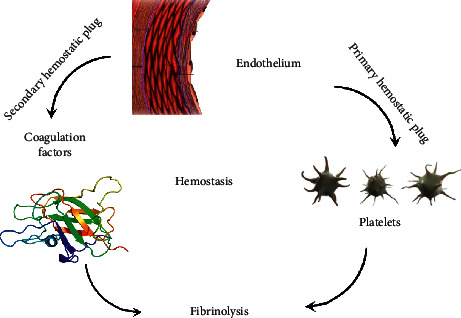
Overview on hemostasis.

The entire process is not over until the fibrin clot formation is inhibited by fibrinolysis. Following bleeding, the clotting mechanism cannot be set perpetual, and the fibrinolytic mechanism of the body inhibits fibrin clot formation once the bleeding has been controlled; thus, maintaining the vascular patency in balance with coagulation (Figure 1) [4]. The central enzyme, plasmin, a serine protease, completes fibrinolysis by breaking down fibrin polymers. Thus formed fibrin degradation products (FDPs) impart feedback inhibition to coagulation [5].

There are three components responsible for the entire clotting mechanism: endothelium, platelets, and coagulation factors [6]. Each one of these components is interconnected on a closely regulated mechanism, and abnormalities in any one of them may result in bleeding disorders. Individuals may either genetically inherit the abnormalities or acquire them during their lifetime. Coagulopathies arise most commonly from inheritance with von Willebrand disease (VWD); however, hemophilia is the most commonly encountered coagulopathy in clinical practice [7]. Bleeding disorders remain one of the most common hematological disorders, and it is important to accurately and timely identify the underlying etiology. This article focuses on the diagnosis of bleeding disorders emphasizing the three components of hemostasis: endothelium, platelets, and coagulation factors.

## 2. Abnormalities in the Primary Hemostatic Plug Formation

The primary hemostatic plug formation is a crucial step in hemostasis. Failure to do so or a defective primary platelet plug formation is first suspected with a prolonged bleeding time (BT) on screening. The Ivy's and Duke's methods are widely applied for estimating BT. The Ivy's method was found superior to the Duke's method in the study by Gobel et al. [8] which can be attributed to the implementation of standardization via constant sphygmomanometer pressure [9]. Physically, the patient may also show bleeding underneath the skin forming petechiae, purpura, or ecchymosis [10].

### 2.1. Endothelial Defect

The endothelium possesses both coagulatory and anticoagulatory functions, and the clotting process is a balance between both of them. Endothelium, on one hand, promotes clotting process by synthesizing the von Willebrand factor (VWF) and platelet adhesion factors, and on the other, impedes platelet adhesion and clotting via prostacyclins, nitric oxides, ecto-ADPase, and tissue plasminogen activator (t-PA) [11–14]. In favor of clotting, the endothelial cells store VWF in a specified organelle, Weibel-Palade bodies [15]. Upon trauma, the membranes of the endothelial cells are disrupted resulting in the exposure of VWF into the blood stream. The VWF is a potent biomolecule that on interaction with the exposed subendothelial collagen and high shear forces has a strong affinity to binding to the glycoprotein Ib (gpIb) of platelets causing platelet adhesion and activation [16]. Inherited defect in VWF is most commonly encountered.

#### 2.1.1. Von Willebrand Disease (VWD)

VWD is caused by the deficiency or the entire absence of VWF [17]. The first indication of VWD is a prolonged BT with normal platelet count. VWD is confirmed by the plasma estimation of VWF antigen (VWF : Ag) testing. Most laboratories employ latex immunoassay and Enzyme-Linked Immunosorbent Assay (ELISA) for the estimation [18]. Clinicians and laboratories should however be aware that VWF : Ag solely cannot distinguish between VWD subtypes. Since VWD types 1 and 3 are quantitative defects, VWF : Ag estimation is suitable in the context, whereas since VWD type 2 is purely a qualitative defect, VWF : Ag may appear normal in most cases.

To accomplish the differentiation of the VWD subtypes, platelet-binding ability (measure of activity) of VWF via ristocetin is a classical yet effective technique where patient's plasma is allowed to interact with normal platelets in the presence of ristocetin. Enhanced activity is a feature in Type 2B VWD. However, the technique holds some drawbacks including low sensitivity, poor reproducibility, higher variation, and false low VWF activity. More recent and reliable techniques involving recombinant platelet glycoprotein gpIb have been developed to overcome the drawbacks of the classical technique. Wild-type gpIb is now available that is more specific and overtakes the sensitivity of the classical technique. The recombinant wild-type gpIb is used as an alternative to platelets, but the presence of ristocetin is still required. In addition, “gain-to-function” mutated gpIb (gpIbM) is also commercially manufactured which even obliviates the requirement of ristocetin in platelet-binding assays [19, 20].

Aforementioned platelet-binding assays in conjunction with VWF : Ag are further a useful tool in the distinction of VWD subtypes. The ratio of the VWD activity and antigen (activity/antigen) is recommended since few patients with type 2 VWD present both normal activity and antigen, with an abnormal ratio. Furthermore, types 1 and 2 can be accurately identified based on the activity-antigen ratio. According to the American Society of Hematology (ASH), the International Society on Thrombosis and Hemostasis (ISTH), the National Hemophilia Foundation (NHF), and the World Federation of Hemophilia (WFH)'s published guidelines, the activity to antigen ratio of less than 0.7 is consistent with type 2 VWD. On the other hand, ratio greater than 0.7 is a feature in type 1 VWD [21].

At the final step, when all the assays have unconvincing results, multimeric analysis of VWF via gel electrophoresis can be performed where the presence or absence of specific multimers can be visually examined [22]. However, multimeric analysis is not incorporated into routine testing due to the costliness and is only performed for specific purposes. Deficiency of larger multimers is associated with more severe form of the disease [23, 24].

### 2.2. Platelet Defect

#### 2.2.1. Thrombocytopenia (Quantitative Defect)

The first step in investigating bleeding disorder is accessing platelet count [25]. Thrombocytopenia involves platelet count below 150,000/mm^3^ and is the most common cause of bleeding [26]. Thrombocytopenia may be caused by both inherited and acquired etiologies [27, 28]. Accessing the platelet count is important prior to initiating the detailed investigation for the bleeding disorder. Clinicians must assure that the patients are not under any thrombolytic and anticoagulation drugs prior investigating [29, 30]. The various causes of thrombocytopenia are displayed in Table 1 [31–36].

#### 2.2.2. Functional (Qualitative) Defect

With a normal platelet count, bleeding may arise when there is any functional anomaly in platelets. Functional defects refer to the defects in adhesion, activation, and aggregation process during platelet plug formation [37]. The Platelet Function Analyzer-100 (PFA-100) is a useful tool in screening the phenomenon of adhesion, activation, and aggregation [38]. It consists of an aperture coated with essential adhesion factors of platelets including collagen, ADP, and epinephrine. When blood is allowed to pass, the platelets bind to these surface agonists, occluding the aperture, and the “closure time” is noted. Prolongation of the closure time indicates defective platelet function [39].

As already discussed above, VWD can impede platelet adhesion. However, there are other platelet factors responsible for adhesion including the glycoprotein Ib (gpIb). Inherited defect in the gpIb results in Bernard-Soulier Syndrome [40]. This condition can be diagnosed by the flow cytometric analysis of the deficient gpIb on platelet surface [41]. Also, the syndrome presents with giant platelets and thus a low platelet count which aid in suspicion [41]. The distinction between VWD and Bernard-Soulier syndrome can be accomplished by the ristocetin-induced platelet aggregation. Both of these conditions will exhibit a defective aggregation and can be distinguished since the addition of normal plasma corrects the defective ristocetin-induced aggregation in VWD and not in Bernard-Soulier syndrome [42]. Ristocetin cofactor assay (RCo) is a similar assay with an advantage of allowing differentiation of VWD subtypes based on the ratio of vWF level and extent of ristocetin activity (vWF : Rco) [43]. Further confirmation can be done using flow cytometry.

Storage deficiency has also been a major concern since it can cause activation defect. Platelets primarily possess three types of granules: alpha and dense granules and lysosomes [44]. The alpha granules store P-selectin, fibronectin, PDGF-4, and TGF-*β*. The dense granules store ADP, histamine, serotonin, and epinephrine. The dense granules are responsible to a larger extent for platelet activation [45]. Storage deficiency can lead to poor platelet aggregation. Platelet aggregation is investigated using an aggregometer which works on the principle that the transmission of light increases as platelets aggregate [46, 47]. Another glycoprotein, activated integrin *α*IIb*β*3, commonly known as gpIIb/IIIa on platelets, is responsible for platelet aggregation by cross-linking platelets with fibrinogen [48]. Defective gpIIb/IIIa on platelets results in inadequate aggregation, and this condition is called Glanzmann's thrombasthenia [49]. Flow cytometric analysis of gpIIb/IIIa aids in diagnosing Glanzmann's thrombasthenia [50]. In brief, any abnormalities in the alpha granules, vWF, fibrinogen, dense granules, Ca^2+^, polyphosphates, and ATP can hinder platelet aggregation. Another important aspect that may hinder normal platelet function is the consumption of antiplatelet effects of drugs including aspirin. Aspirin acts by the acetylation of the platelet cyclooxygenase enzymes leading to diminished formation of prostaglandins and thromboxane from arachidonic acid, ultimately hindering platelet aggregation. Generally, platelet aggregation is normalized after 96 hours of the latest aspirin ingestion [51]. Furthermore, the presence of a high amount of FDPs competes against fibrinogen and binds to platelets inhibiting platelet aggregation [52].

#### 2.2.3. The ISTH-BAT Score in Suspected Inherited Bleeding Defect

In 2010, the International Society of Thrombosis and Hemostasis (ISTH) proposed a checklist as a bleeding assessment tool (BAT) for the screening of inherited bleeding disorder [53]. The checklist assesses the potentiality of an underlying bleeding disorder via symptoms and history of the patient under 14 distinct categories. The categories include excess bleeding history from skin, joints, nose, oral cavity, dental procedures, gastrointestinal and urogenital tracts, excessive menstrual bleeding, past surgeries, and transfusion requirements [54]. The main aim of the assessment tool is to lower the need of laborious laboratory tests. The ISTH-BAT scores have shown significant associations in underlying bleeding disorders in various studies. In a study by Adler et al. [55] conducted in 555 patients with suspected bleeding disorder, 288 (51.9%) patients were diagnosed with a bleeding disorder with the highest frequencies being observed in platelet function defects and vWD. However, Fasulo et al. [56] showed contrary reports in his study. The ASH Clinical News in 2019 published that ISTH-BAT is validated for the suspicion of vWD, and its use for platelet function defect has not been investigated [57]. Pathare et al. [58] also presented supportive evidence of significant correlation of the ISTH-BAT with vWF : Ag measurement. Currently, the topic is still a matter of research, and no universal validation has been announced.

### 2.3. Coagulation Factor Defect

A majority of coagulation factors except fibrinogen are not involved in the primary hemostatic plug formation. Fibrinogen is a protein produced by the liver and is required for the cross-linking of platelets during the aggregation process. Hence, states where the liver function is compromised can greatly influence plasma fibrinogen concentration [59–61]. Two types of fibrinogen assay are now widely used. One based on the thrombin time method and another based on latex agglutination. However, thrombin time method has an advantage that it estimates only the functional fibrinogen in the plasma while the latex-based assay estimates all functional as well as nonfunctional fibrinogens which may be encountered in dysfibrinogenemia. In dysfibrinogenemia, there are high chances that the latex-based assay may overestimate the functional fibrinogen by including the nonfunctional as well [62, 63]. Estimation of FDPs and specifically D-dimer can be performed as these can impede coagulation by competing against the fibrinogen [6]. FDPs are the indication of fibrinolysis and are significantly elevated in disseminated intravascular coagulation (DIC) (Fibrin Degradation Products [64]).

## 3. Abnormalities in the Secondary Hemostatic Plug Formation

Affected secondary hemostatic plug formation is mainly due to the deficiency of coagulation factors and has less to do with the endothelium and platelets. The first features are seen during the screening tests as prothrombin time (PT) or activated partial prothrombin time (APTT) or both shows abnormal results. Thrombin time (TT) is specific to screening for thrombin formation. Clotting time (CT) is also performed by most of the laboratories but may mislead the diagnosis since abnormal results are obtained only after the factor deficiency is huge that its plasma activity is <5% [65]. While most often the abnormal results arise due to one or multiple factor deficiencies, circulating inhibitors result it to a lesser extent [66]. Clinicians should however remember that it is important to rule out the liver involvement in cases of prolonged PT or APTT. The liver is responsible for the synthesis of coagulation factors [67], and chronic liver diseases result in decreased synthesis of coagulation factors. Assessment of the liver function tests (LFTs) should be performed first prior to in-depth investigation of a coagulopathy [68, 69]. Another thing to remember here is that when LFTs are normal, vitamin K deficiency might be the reason. Vitamin K is required for the gamma-carboxylation of the coagulation factors so that they can function normally. Assessment of PT/APTT after vitamin *K* supplementation can also be performed [70–72]. PT is more sensitive to vitamin *K* deficiency since the factor VII which is the initiating factor of the extrinsic pathway is vitamin *K* dependent [73]. One more important fact to know is that consumption of aspirin will also prolong these tests [74]. These screening tests should be performed at least twice before starting the treatment to ensure that the abnormal results are not due to preanalytical, analytical, and postanalytical errors. If these parameters are normal, then the clinician can proceed to the in-depth investigation by confirmatory testing.

### 3.1. Identification of the Etiology

The first step is to identify whether the abnormal results are due to factor(s) deficiency or presence of inhibitor(s). The PT/APTT mixing tests are commonly performed for the screening. When normal plasma is mixed with the test plasma, then the factors are supplied by the normal plasma which is supposed to correct the prolonged test. If there are inhibitors present, even the addition normal plasma cannot correct it and the test remains prolonged [75, 76]. However, confusion might arise in case of delayed acting inhibitors. In the case, the test initially shows correction and points out underlying factor deficiency but later is prolonged again due to the action of delayed acting inhibitor [77]. That is why testing mixed test plasma after 2 hours of incubation is important to detect these inhibitors.

### 3.2. Confirmatory Tests of the Secondary Hemostatic Defects

Once the etiology is identified, the absence of the specific factor of the presence of the inhibitor can be tested. Factor assays are widely performed in routine laboratories today. Factor VIII deficiency is the most prevalent which is termed as hemophilia A [78]; [79]. It is an X-linked disorder that predominantly affects the males with a global prevalence of 1 in 5000 male births [80]; [81]; [17]. In von Willebrand disease, factor VIII concentration is low since a fraction of it remains bound to VWF which protects from destruction in circulation. A less common disease is hemophilia B which is due to factor IX deficiency and is seen in approximately 1 in 25000 male births [82]. Both of these diseases are suspected by isolated prolongation of PT. Apart from these, factor XI, V, and X deficiencies may be encountered but are very occasional [83, 84]. Most of the laboratories utilize factor-deficient plasma reference graph method that measures factor activity and has shown better performance [85].

Test plasma sometimes present normal findings with normal factor activity and absence of inhibitors. However, prolonged PT/APTT is obtained. In such cases, hypofibrinogenemia is the cause which results from DIC or chronic liver diseases [86]. As discussed earlier, the thrombin time method for fibrinogen as proposed by Clauss is preferred since it provides the measure of functional fibrinogen. Again, the thrombin time method for fibrinogen may be prolonged even in normal fibrinogen concentrations when heparin is present in the blood which inhibits thrombin formation [87]. The reptilase time has been proposed that can successfully distinguish between hypofibrinogenemia and heparin. Reptilase is the venom from the snake *Bothrops atrox* that can interfere with the action of heparin [88]. Alike mixing tests discussed above, the test plasma is mixed with reptilase and the PT/APTT is retested. In case of heparin, the tests which were previously prolonged are now corrected to normal reference range. The tests remain prolonged in case of dysfibrinogenemia, hypofibrinogenemia, or afibrinogenemia [89].

Various types of inhibitors are identified that are responsible for the prolonged coagulation tests and are categorized into specific and nonspecific inhibitors [90].; [91]. Anti-factor VIII antibody is the most common specific inhibitor and is likely to be present in hemophilia A and in individuals receiving multiple transfusions [92–94]. Lupus anticoagulant is a nonspecific inhibitor which is an antiphospholipid antibody [95]. Phospholipids confer a negatively charged surface on the platelets and bind coagulation factors via calcium [96]. While lupus anticoagulants present paradoxically prolonged coagulation tests on one hand, a strong risk of arterial and venous thrombosis exists on the other [97]. Diluted Russell Viper's Venom Time (dRVVT) is nowadays popular in detecting lupus anticoagulant. The test is not affected by factor VIII or IX inhibitors and generates sensitive and specific results [98]. Lupus anticoagulant is commonly encountered in systemic lupus erythematosus (SLE), autoimmune disorders, malignancies, and administration of phenytoin, quinine, procainamide, chlorpromazine, and hydralazine [99].

## 4. Disseminated Intravascular Coagulation (DIC): An Overlapping Presenter

**Figure 2:**
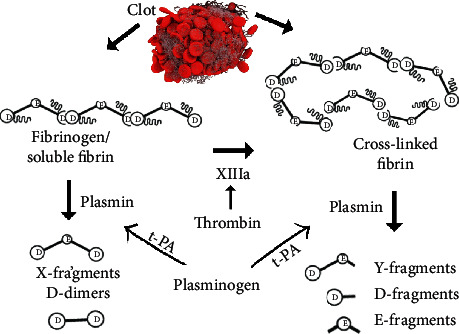
Formation of different FDPs from a blood clot.

DIC is a systemic manifestation of underlying conditions that result in consumption of platelets by extensive activation of the coagulation cascade [100]. Some of the common triggers of DIC are brain trauma, ongoing hemolysis, blood stream infection, pre-eclampsia, other hypertensive diseases complicating pregnancy, snake bite, acute promyelocytic leukemia, and recent surgery [101, 102]. DIC presents with abnormalities in both primary and secondary hemostatic plug defects due to consumption of platelets as well as coagulation factors. When tests for both categories are abnormal, DIC can be suspected given a history of trigger [103]. Microangiopathic hemolytic anemia is a common manifestation of DIC due to RBC injury by microclots in vessels [104]; [105]. Peripheral blood film examination reveals fragmented red cells “schistocytes” in large numbers [106]. That's why, a peripheral blood film is important to examine at the initial investigation stage of coagulopathies so that indication of DIC can be obtained. Another distinguishing feature of DIC is the elevated blood fibrin degradation products (FDPs) due to destruction of the huge amount of fibrin formed (“Fibrin Degradation Products [64]). D-dimer is the end product of fibrin destruction and is widely measured using monoclonal antibody latex agglutination [107, 108]. Practically, all FDPs do not end as d-dimers, and some of them remain as X and Y fragments Figure 2, especially in early DIC and the estimation of d-dimer alone in such situation can underestimate the extent of DIC [90]. Occasionally, soluble fibrin monomers may arise which can be detected by the ethanol and protamine sulfate gelation test, and a positive gelification is suggestive of intravascular coagulation [109, 110].

## 5. Viscoelastic Hemostatic Assays in Diagnosing Bleeding Disorders

**Figure 3:**
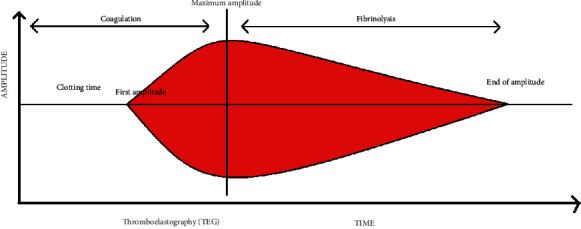
A normal thromboelastography (TEG).

Studying the viscoelastic property of blood has been gaining popularity in recent days. As blood starts to coagulate, there appears an increase in the viscoelasticity, and it is reduced as fibrinolysis sets in and the clot is eliminated. Some laboratories utilize the principle as thromboelastography (TEG) [111, 112]. TEG is however not a novel technique and was developed in 1948 [112]. The architecture consists of a cup and a pin suspended into the cup. The cup oscillates across the pin. The working design is similar to a seismograph. In TEG, the test blood is filled in the cup and is oscillated across the pin suspended vertically into the blood. As clot formation begins, it adheres to the pin and makes it move along with it [113]. As the pin moves, a graph is generated [114] as shown in Figure 3.

As the clot gets strengthened, the amplitude increases proportionally. The time from the start of oscillation to the appearance of first amplitude is the clotting time. The maximum amplitude is when the clot formation is completed. As fibrinolysis sets in, the clot's adherence to the pin is reduced and so is the amplitude. When fibrinolysis is completed, the pin is again motionless and the amplitude is zero. The time between the highest amplitude and its nullification is the time for complete fibrinolysis [115]. Since the test possesses a rapid turnaround time of 15 to 20 minutes, it can be used as a point-of-care testing. Rotational thromboelastometry (ROTEM) is also a similar procedure which is used. The difference between TEG and ROTEM is that the cup is the one that oscillates across the pin in TEG while in ROTEM, the pin oscillates and the cup is fixed [116–118].

**Figure 4:**
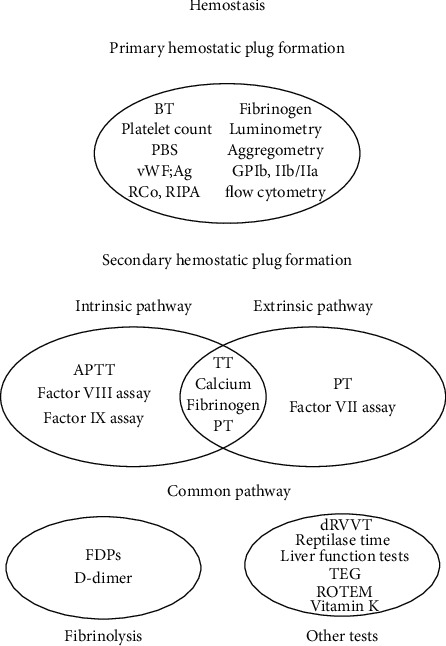
A schematic summary of different coagulation tests in accordance to the steps in hemostasis.

A schematic summary of different coagulation tests in accordance to the steps in hemostasis is shown in Figure 4).

## 6. Conclusion

Bleeding disorders are one of the most prevalent hematological disorders worldwide. The article simplifies the diagnosis of the vague term “bleeding disorder” into three basic yet vital components, including endothelium, platelets, and coagulation factors. It is important to understand the physiology of each of the subgroups and know how an individual defect affects the entire coagulation system of the body. When investigation is carried out in this perspective, there are few chances to miss the definitive diagnosis. Viscoelastic methods allow the study of complete physiology of blood clotting of the patient including fibrinolysis. Interpreting bleeding disorders can be erroneous when the defects in these components are not properly and entirely investigated. This perspective enables clinicians and laboratories to accurately identify the defect and guides to the selection of specific treatment options for the patient.

## Figures and Tables

**Table 1:** Table displaying various causes behind thrombocytopenia.

Increased destruction	Decreased production
*Immune*	*Hereditary*
Idiopathic thrombocytopenic purpura systemic lupus erythematosus drugs: heparin, penicillin, quinidine, quinine infections: HIV, malaria post-transfusion purpura, and neonatal alloimmune purpura.	Fanconi's anemia and Wiskott-Aldrich syndrome

*Nonimmune*	*Acquired*
Disseminated intravascular coagulation, thrombotic thrombocytopenic purpura, hemolytic uremic syndrome, and giant hemangioma	Aplastic anemia, megaloblastic anemia, hematological malignancies, thrombolytic drugs, and viral infections

*Others*	
Dilutional thrombocytopenia following massive blood transfusion, increased sequestration in hypersplenism, Bernard-Soulier syndrome, von Willebrand disease type 2B, chemotherapy-induced thrombocytopenia (CIT), and radiation-induced thrombocytopenia (RIT).	

## Data Availability

All data used to support the findings of the study can be obtained within the article.
